# Identification and Characterization of Two Functionally Unknown Genes Involved in Butanol Tolerance of *Clostridium acetobutylicum*

**DOI:** 10.1371/journal.pone.0038815

**Published:** 2012-06-29

**Authors:** Kaizhi Jia, Yanping Zhang, Yin Li

**Affiliations:** Institute of Microbiology, Chinese Academy of Sciences, Beijing, China; Centre National de la Recherche Scientifique, France

## Abstract

Solvents toxicity is a major limiting factor hampering the cost-effective biotechnological production of chemicals. In *Clostridium acetobutylicum*, a functionally unknown protein (encoded by SMB_G1518) with a hypothetical alcohol interacting domain was identified. Disruption of SMB_G1518 and/or its downstream gene SMB_G1519 resulted in increased butanol tolerance, while overexpression of SMB_G1518-1519 decreased butanol tolerance. In addition, SMB_G1518-1519 also influences the production of pyruvate:ferredoxin oxidoreductase (PFOR) and flagellar protein hag, the maintenance of cell motility. We conclude that the system of SMB_G1518-1519 protein plays a role in the butanol sensitivity/tolerance phenotype of *C. acetobutylicum*, and can be considered as potential targets for engineering alcohol tolerance.

## Introduction

The toxicity of organic solvents to microorganisms is a major limiting factor hampering the cost-effective biotechnological production of solvents [1], [2]. Alcohol is a class of solvents, most of which can be produced by microorganisms. The alcohol tolerance of microorganisms is a very complex phenotype, which is known to be affected by stress proteins, transcription factors, efflux pumps, small-molecule chaperones, compatible solutes, membrane composition, and energy metabolism [3].

On the other hand, alcohol can be used as an anesthetic. The anaesthetic effect was initially ascribed to the perturbation of cell membrane [4], [5], [6]. However, the concentration of alcohol used clinically might be too low to induce the perturbation of cell membrane in animal cells [7]. Another theory proposes that protein kinase C (PKC) mediates alcohol toxicity. PKC is an important signal transduction protein with cysteine-rich zinc finger subdomains C1A and C1B. PKC was involved in the sensitivity to alcohol by non-specific interaction with alcohol in diacylglycerol/phorbol ester-binding subdomains of C1A and C1B [8]. Alcohol binding sites are discretely presented within C1A and C1B, and two of these binding sites were located in the vicinity of the phorbol binding loops, suggesting the modulating function of alcohol [5], [8], [9]. In this way, zinc finger domain plays a vital role in mediating the effects of alcohol on animal cell.

In various animal cells, cysteine-rich zinc finger subdomains of PKC interacting with alcohol are highly conserved (Figure S1). Zinc finger structures are found in many microorganisms and known to perform important regulation tasks during microbial physiological process [10], [11]. Therefore we examined the hypothesis that a regulator with possible alcohol interacting domain might be present in microorganisms and involved in butanol tolerance.

*Clostridium acetobutylicum* is an important producer of solvents (acetone, ethanol and butanol). Among these products, butanol is the most toxic as it reduces cell growth by 50% at a concentration of 7–13 g/L [12], [13]. Besides continuous gas stripping, engineering microbial butanol tolerace is another important strategy for reducing or eliminating butanol toxicity [14]. Butanol tolerant mutants could be obtained through two strategies: random approach which include the random mutagesis [15], genome shuffling [16], or genomic library enrichment [17], and rational design which include the overproducing cyclopropane fatty acid synthase (changing the lipid composition) [18], class I stress response operon *groESL* [12], the master regulator of sporulation Spo0A [19]. However, little is known about whether negative regulatory factors were involved in butanol tolerance in *Clostridium*. To test the above described hypothesis, potential candidate genes were identified in the genome from bioinformatics analysis. The functions of the candidate genes were then characterized.

## Results

### Rationale

As alcohol interacting regions are highly conserved in animal cells (Figure S1) and these cysteine-rich zinc finger domains were found in many sequenced microbes, we propose that a protein containing this conserved region might function in modulating butanol tolerance in *C. acetobutylicum*. In order to identify such possible proteins, the first step is to filter the proteomic information of *C. acetobutylicum* through a series of criteria until potential candidate proteins are obtained. These candidate proteins are expected to share structural and sequence similarity to the regulating region of PKC and possess the alcohol binding sites.

### Identification of SMB_G1518-1519 as Potential Target Mediating Butanol Tolerance

PKC superfamily contains 8 types of isomers, the mechanism for PKC isomers α and δ interacting with anesthetics has been extensively studied [8], [9], [20]. Alcohol binding sites are discretely presented in the C1 domain, which consists of a tandem repeat of highly conserved cysteine-rich zinc finger subdomains C1A and C1B. We scanned the proteome of *C. acetobutylicum* DSM 1731 (its whole genome sequence shares 99% similarity to that of the type strain *C. acetobutylicum* ATCC 824 [21]) using the highly conserved alcohol interacting region (residues 159–208 of C1A and 231–280 of C1B) as query protein sequences [8]. The NCBI blast generated 11 candidate proteins, which showed over 30% similarity to the conserved butanol interacting region in PKC. Only one protein, encoded by SMB_G1518 (annotated as CAC1493 in the genome of type strain *C. acetobutylicum* ATCC 824), contains Zn-finger DNA-binding domain, and the potential butanol binding sites such as Tyr, Lys and Glu also appear to be dispersed throughout the conserved region. SMB_G1518 is located in a two-gene operon together with SMB_G1519 (annotated as CAC1494 in the genome of *C. acetobutylicum* ATCC 824) [21]. The stop codon of SMB_G1518 overlaps with the start codon of SMB_G1519, suggesting that their expression must be cotranslationally coupled. Therefore, we predicted that these two genes are involved in the same physiological process in *C. acetobutylicum*.

### Inactivation of SMB_G1518-1519 Increased the Tolerance to Butanol

As SMB_G1518 contains cysteine-rich zinc finger domain putative interacting with alcohol, inactivation of SMB_G1518, SMB_G1519, and SMB_G1518-1519 is expected to make the mutants less sensitive to butanol. To test this hypothesis, we inactivated SMB_G1518 and SMB_G1519, respectively, by using the ClosTron system based on group II intron retrotransposition. The genotypes of the resulting mutants DC93 and DC94 were confirmed by sequencing PCR products and southern blot (Figure S2). Construction and confirmation of the SMB_G1518-1519 deletion mutant DDC14 have been conducted in a previous study [22].

**Figure 1:**
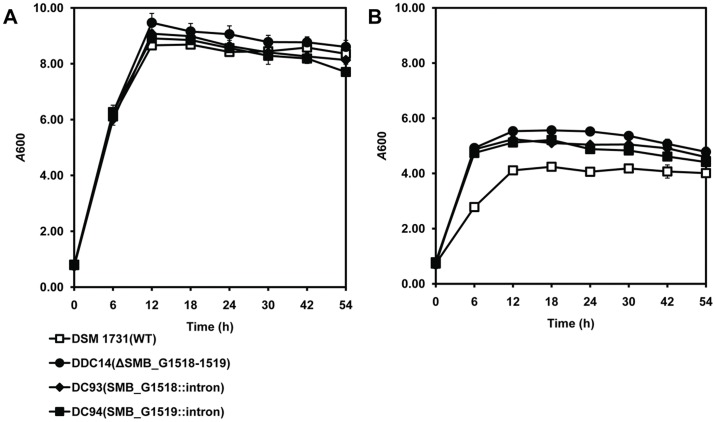
Growth profiles for DSM 1731 and its deletion or disruption mutants. A) Growth profiles under normal condition. B) Growth profiles under 1% butanol stress.

**Figure 2:**
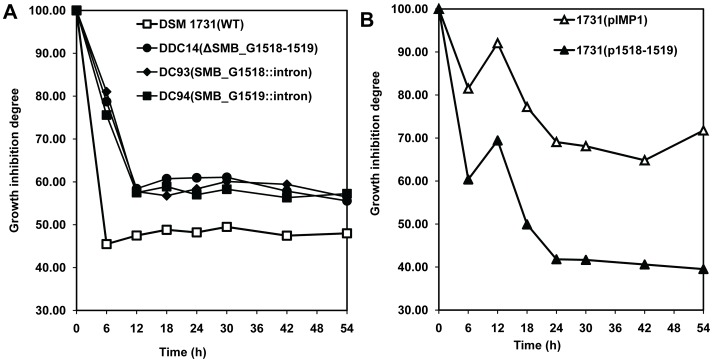
Diagram of growth inhibition. A), DSM 1731 and its deletion or disruption mutants DDC14, DC93 and DC94. B) 1731(pIMP1) and 1731(p1518-1519). The growth inhibition level was determined by using the following formula: [(*A*600)+BuOH/*A*600]×100, *A*600+BuOH is referred to cell density in the presence of butanol.

Cell growth *A*600 has been regarded as one of the most sensitive indicator for assessing butanol tolerance of *C. acetobutylicum* [23]. The deletion or disruption mutants DDC14, DC93, DC94, and their parent strain DSM 1731 were subjected to 1% (vol/vol) butanol challenge when *A*600 reached 0.75±0.05 (mid-exponential growth), followed by measuring the subsequent growth and calculating the growth inhibition degree (Figure 1). Under normal condition, there was no difference in cell densities among the mutant strains and the parent strain (Figure 1A). Not surprisingly, the addition of 1% butanol significantly inhibited the growth of all strains. However, the deletion mutant DDC14 and the disruption mutants DC93 and DC94 grew faster and achieved over 70% higher final *A600* than that of the wild type strain DSM 1731 after 6 h cultivation (Figure 1B). This suggests that SMB_G1518-1519 encoding proteins play a major role in regulating butanol tolerance. Disruption mutant DC94, in which only the SMB_G1519 gene was inactivated, has the same phenotype than strains DDC14 and DC93. It indicated that a polar effect on expression of SMB_G1518 can be ruled out since SMB_G1519 is located downstream in the operon. Comparison of the growth inhibition degree showed there is no significant differences among the deletion mutant DDC14 and the disruption mutants DC93 and DC94 (Figure 2A), suggesting that the biological function of SMB_G1519 is closely related to SMB_G1518 so as inactivation of single or both genes all contributed to the increased butanol tolerance upon butanol challenge.

### Functional Identification of SMB_G1518-1519 by its Overexpression

To prove that SMB_G1518-1519 encoding proteins were involved in butanol toxicity, the strain with overexpression of SMB_G1518-1519 was constructed. To minimize the potential polar effect of gene overexpression, the DNA fragment containing SMB_G1518-1519 and their own promoter was cloned into an expression vector pIMP1 (copy number of 8) [24]. Thus, gene overexpression is achieved solely by increasing the copy number of SMB_G1518-1519. Quantitative reverse transcription-PCR showed that the transcript levels of SMB_G1518-1519 in overexpression strain 1731(p1518-1519) increased by 89 fold as compared to that of the control strain 1731(pIMP1) (Figure S3A). Further semi-quantitative PCR result also proved that SMB_G1518-1519 exhibited much higher transcriptional levels in overexpression strain 1731(p1518-1519) than in plasmid control strain 1731(pIMP1) (Figure S3B). These results together with the phenotypic analysis of disruption and deletion mutants indicated that SMB_G1518 and SMB_G 1519 were coexpressed.

**Figure 3:**
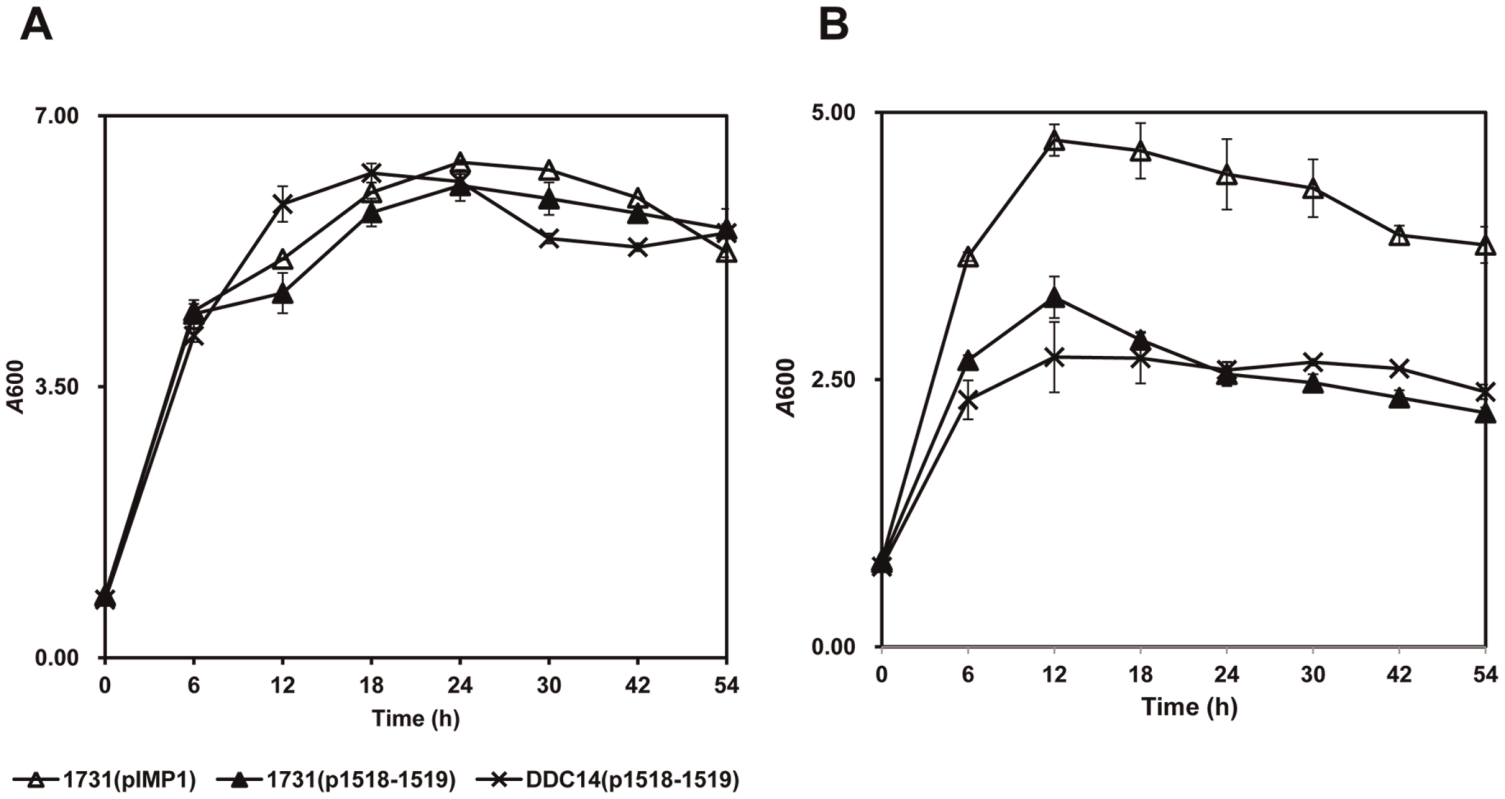
Growth profiles for 1731(pIMP1), 1731(p1518-1519) and DDC14(p1518-1519) . A) Growth profiles under normal condition. B) Growth profiles under 1% butanol stress.

Under normal condition, overexpression of SMB_G1518-1519 in DSM 1731 did not alter the growth pattern as compared to the control strain 1731(pIMP1) (Figure 3A). However, when both strains were subjected to 1% butanol challenge, the growth of strain 1731(p1518-1519) was significantly inhibited as compared to that of the control strain 1731(pIMP1), which indicates SMB_G1518-1519 encoding proteins are growth inhibitors in response to butanol stress (Figure 3B). 1731(p1518-1519) exhibited more severe growth inhibition than its control 1731(pIMP1), this indicates that SMB_G1518-1519 encoding proteins may be butanol stress proteins (Figure 2B).

The growth pattern of DDC14(p1518-1519) is similar to overexpression strain 1731(p1518-1519) under normal condition or butanol stress, suggesting the introduction of p1518-1519 (copy number of 8) into SMB_G1518-1519 deletion mutant DDC14 made the host sensitive to butanol stress in view to the overexpression of SMB_G1518-1519 (Figure 3A and B).

### Fermentation Products Analysis

**Figure 4:**
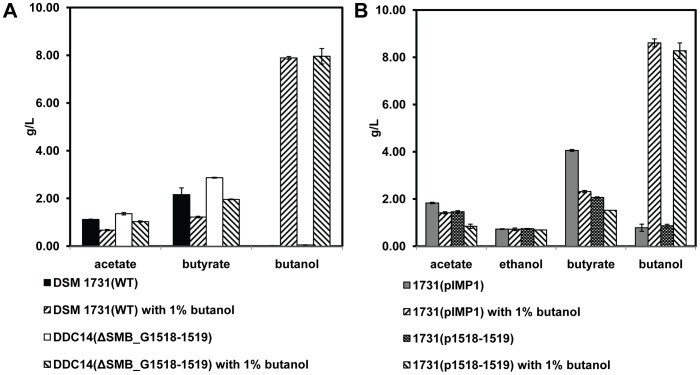
The analysis of main fermentation products after 50% of growth inhibition degree was achieved. A) The analysis of main fermentation products after DSM 1731 and its deletion mutant DDC14 were treated by butanol for 6 h. B) The analysis of main fermentation products after the plasmid control strain 1731(pIMP1) and overexpression strain 1731(p1518-1519) were treated by butanol for 18 h.

To rule out the influence of fermentation products on the growth, fermentation products of deletion mutants DDC14, overexpression strain 1731(p1518-1519) and their respective controls were analyzed when 50% of growth inhibition degree was achieved by 1% butanol treatment (Figure 4). Under normal condition or butanol stress, higher concentration of acetate, butyrate and butanol were found to be accumulated in the broth culture of deletion mutant DDC14 than that of its control strain DSM 1731 (Figure 4A). Relative to plasmid control strain 1731(pIMP1), lower concentration of acetate and butyrate and no significant variation in amount of butanol were detected in culture inoculated with overexpression strain 1731(p1518-1519) (Figure 4B). Acetone was not detected in the broth cultures of all these strains. Ethanol was only detected in the cultures inoculated with overexpression strain 1731(p1518-1519) and its plasmid control 1731(pIMP1) because of the addition of Erythromycin which is solved in ethanol (Figure 4B). All these results indicated that SMB_G1518-1519 encoding proteins instead of the variation of fermentation products impacted the tolerance to butanol in *C. acetobutylicum*.

### Proteomic Analyses of *Clostridium acetobutylicum* SMB_G1518-1519 Mutants and Overexpression Strain

**Figure 5:**
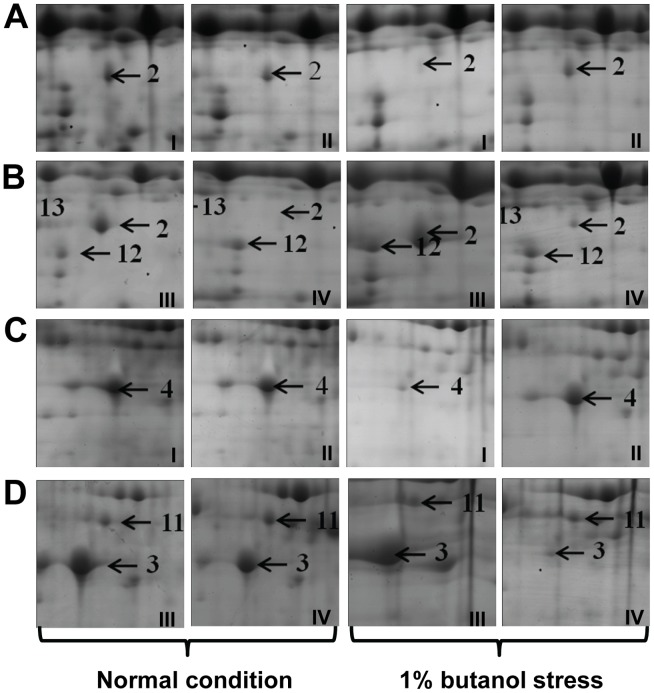
Close-up views of the protein spots with differential expression. I, DSM 1731; II, DDC14; III, 1731(pIMP1); IV, 1731(p1518-1519). A) The differentially expressed PFOR in DSM1731 and DDC14. B) The differentially expressed PFOR in 731(pIMP1) and 1731(p1518-1519). C) The differentially expressed Hag in DSM1731 and DDC14. D) The differentially expressed Hag in 731(pIMP1) and 1731(p1518-1519).

The observation that the function of SMB_G1518-1519 was closely related with butanol tolerance prompted an investigation of the biological mechanism on butanol tolerance. SMB_G1518-1519 encoding proteins were thought to be involved in the regulation of butanol tolerance through protein-protein interaction due to that Zn finger located in their N-terminal end [10]. Therefore, the cytoplasmic proteomes of the overexpression strain 1731(p1518-1519), the deletion mutant DDC14, and their respective controls, in the absence or presence of 1% butanol stress were compared in order to explaining the reason for the enhancement of butanol tolerance by inactivation of SMB_G1518-1519 (Figure S4, S5, S6, S7). The resulting proteomic data were normalized by using the proteomes of DSM1731 or 1731(pMP1) under normal condition as standardized map. Proteins increased over 2-fold differential expression, in all biological replicates of at least one treatment, were listed in Table 1. Most of these proteins were involved in the carbohydrate metabolism, cell motility, chaperone and fatty acid synthesis. Two proteins, pyruvate:ferredoxin oxidoreductase (PFOR) involved in pyruvate metabolism and Hag involved in cell motility, were found differentially expressed in both deletion mutant DDC14 and overexpression strain 1731(p1518-1519) (Figure 5).

**Table 1:** Functional classification of different proteins.

		Ratio					Ratio		
Spot	Protein	DDC14	DSM 1731 (1% butanol)	DDC14 (1% butanol)	Spot	Protein	1731(p1518-1519)	1731(pIMP1)(1% butanol)	1731(p1518-1519)(1% butanol)
	Carbohydrate metabolism					Carbohydrate metabolism			
1	Isocitrate dehydrogenase	0.8	3.6	1.5	1	Mixture: Glucose-6-phosphate isomerase + Thiamine biosynthesis protein ThiC	1.6	−0.8	1.2
2	**Pyruvate:ferredoxin oxidoreductase**	**0.8**	**−2.7**	**1.1**	2	**Pyruvate:ferredoxin oxidoreductase**	**−3.6**	**1.2**	**−2.4**
3	DTDP-D-glucose 4,6-dehydratase	0.6	−1.3	−3.1					
	Cell motility					Cell motility			
4	**Hag**	**0.7**	**−3.8**	**0.6**	3	**Hag**	**−1.6**	**0.8**	**−5.8**
	Chaperone					Chaperone			
5	GroEL	1.0	2.4	0.5	4	Phage shock protein A	1.3	1.2	2.0
					5	HSP18	−3.5	1.3	1.8
					6	HSP18	−8.2	1.9	1.0
					7	GrpE	1.0	1.8	2.2
	Amino acid synthesis					Fatty acid synthesis			
6	Phosphoribosylaminoimidazole-succinocarboxamide synthase	0.7	−0.9	−3.6	8	Acyl-ACP thioesterase	0.3	0	2.5
					9	3-oxoacyl-(acyl-carrier-protein) synthase I	1.4	−0.5	2.4
						Membrane transport			
					10	F0F1-type ATP synthase alpha subunit	3.3	−2.8	0.6
						Others			
					11	Cell division GTPase FtsZ	−0.9	−0.9	−4.7
					12	3-hydroxybutyryl-CoA dehydrogenase(BHBD)	−0.6	−5.5	0.3
					13	Metal-dependent hydrolaseof the beta-lactamase	0	6.8	1.9

Note: The protein levels in DSM 1731 and 1731(pIMP1) under normal condition were used as the basis for comparison, respectively. The upregulation of fatty acid synthesis enzymes (acyl-ACP (acyl-carrier-protein) thioesterase and 3-oxoacyl-ACP synthase I) is more likely to be an indicator reflecting cell damage from butanol stress. The upregulation of HSPs is regarded as a common response to butanol stress, but not related with the function of SMB_G1518-1519.

PFOR catalyzes the coenzyme A (CoA)-dependent oxidative decarboxylation of pyruvate. Under normal conditions, no significant variation in expression level of PFOR was detected in the deletion mutant DDC14 and its control DSM 1731 (Table 1, Figure 5A). Butanol stress made this protein downregulate 2.7-fold in the wild type strain DSM 1731 while had no significant effect on that of the deletion mutant DDC14 (Table 1, Figure 5A). This indicates that the expression of PFOR responded to butanol stress, it is regulated by SMB_G1518-1519 encoding proteins. Overexpression of SMB_G1518-1519 downregulated PFOR 3.6- and 2.4-folds under normal condition and butanol stress, which indicates that SMB_G1518-1519 encoding proteins constitutively inhibited the expression of PFOR (Table 1, Figure 5B).

**Figure 6:**
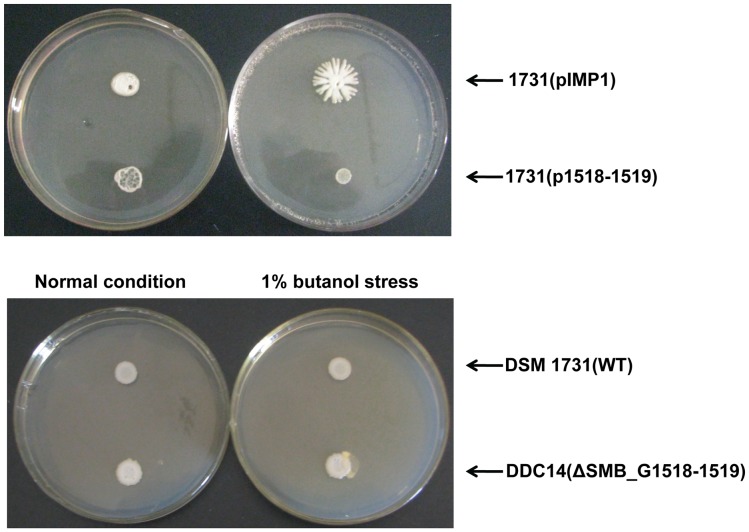
Overexpression of SMB_G1518-1519 influenced the motilities of *C. acetobutylicum*.

Hag makes up the flagellum basal structure flagellin which assembles flagellum filament. Under normal condition, the deletion or overexpression of SMB_G1518-1519 had no significant effect on the expression level of Hag (Table 1, Figure 5C). While Hag was significantly downregulated for 3.8 and 5.8-folds in the wild type strain DSM 1731 and overexpression strain 1731(p1518-1519) under butanol stress, which suggests that SMB_G1518-1519 encoding proteins are likely to be repressors of Hag in response to butanol stress (Table 1, Figure 5C and D). The downregulation of Hag may make flagellar filament shorter and further affect the cell motility. Motility is defined as the ability of cells to spread away from the edge of inoculation point driven by flagella, which is observed on media solidified with agar [25]. In our study, different strains with varied tolerance to butanol were spotted onto CGM plates. Under normal condition, the pattern of colony spread was similar (Figure 6). Upon 1% butanol stress, no obvious difference in mobility degree was observed in DSM 1731 and its mutant DDC14 (Figure 6). The plasmid control strain 1731(pIMP1) moved with a much stronger ability, while the spotted culture of overexpression strain 1731(p1518-1519) remained at the inoculation point, suggesting that overexpression of SMB_G1518-1519 impaired motility under butanol stress (Figure 6).

## Discussion

Alcohol toxicity was regarded as one of the key problems associated with the fermentative production of alcohol [16], [26]. Many investigators proposed the development of alcohol-tolerant solventogenic strains together with in situ recovery processes as a potential solution [26], [27], [28]. In view to that alcohol tolerance is a complex and multigenic phenotype, engineering transcriptional regulators would be a better choice for the enhancement of alcohol tolerance. Two positive regulators spo0A and CAC1869 have been identified thus far in increasing butanol tolerance. In this study, a novel regulational factor encoded by SMB_G1518 was found to weaken alcohol tolerance. SMB_G1518 and SMB_G1519 are all involved in regulating butanol tolerance, as disruption of either one or deletion of both genes resulted in similar phenotypes. The expression of SMB_G1518-1519 decreased the tolerance to butanol, downregulated the production of PFOR and Hag, result in the reduced mobility. However, inactivation of SMB_G1518 and its downstream gene SMB_G1519 or both genes made the strains grow faster, even increased final *A*600 by over 70% when these mutants suffered from 1% butanol stress at the initial stage. This indicated SMB_G1518- G1519 encoding proteins may be negative regulators involved in butanol tolerance and used as ideal targets for engineering alcohol tolerance.

The variation of flagellum components especially Hag can result in the change in motility [29], [30]. This process is regulated by aconitases acting as iron and oxidative stress-responsive posttranscriptional regulators in *Salmonella enterica*serovar Typhimurium LT2 and CsrA acting as carbon storage regulator in *Bacillus subtilis* [30], [31]. In our study, the existence of SMB_G1518-1519 downregulated Hag expression and overexpressing it reduced the cell mobility significantly upon butanol stress. We therefore concluded that SMB_G1518-1519 encoding protein repressed mobility by inhibiting Hag production. Strain DSM 1731 containing one copy of SMB_G1518-1519 downregulated Hag expression significantly, but its motility ability was not altered. This might be related to the content of Hag in *C. acetobutylicum. C. acetobutylicum* DSM 1731 belongs to the multiple-flagellin systems because it possesses 4 flagellin genes (*hag*, SMB_G1580, *flaB3*, SMB_G2236) and encodes four flagellins approximately of 30 kDa. A typical feature of multiple-flagellin systems is that they have redundant flagellins [29]. Therefore, significant inhibition of cell motility was not observed until the amount of flagellin decreased below a threshold level, which subsequently led to shortened filament lengths [29]. In microorganisms, motility confers cell with antimicrobial resistance [32], [33], while our work extends it to solvent resistance. Introducing expression plasmid pIMP1 into DSM 1731 enhanced not only its tolerance to butanol, but also the motility ability; overexpression of SMB_G1518-1519 impaired the motility ability and the resistance to butanol. These results also indicated that the mechanism for host-plasmid interactions in *Clostridium acetobutylicum* was contradictory to traditional knowledge which attributed it to that the presence of a plasmid represents a metabolic burden and a cellular stress [34]. Transcriptional analysis indicated that the presence of plasmid pIMP1resulted in increased levels of HSPs and altered levels of carbon metabolism genes [12]. Our study showed that it lead to increased levels of Hag which function in cell motility. Recent evidence suggests that host-plasmid interactions are closely related to the alteration of cellular regulatory status [34].

The significance of this work is the discovery of two unknown genes SMB_G1518 and SMB_G1519. Their functional identification unraveled at least part of the complex physiological mechanism of alcohol tolerance in prokaryotes. Zinc finger protein was found to be existed in many sequenced microbial strains and may have a chance to be involved in alcohol tolerance like SMB_G1518 encoding protein. If so, it can be regarded as potential target for engineering microbial alcohol tolerance.

## Materials and Methods

### Bacterial Strains, Plasmids, and Growth Conditions

Plasmids and strains used in this study are listed in Table 2. *E. coli* strains were grown aerobically at 37°C in LB broth. *C. acetobutylicum* strains were grown anaerobically at 37°C in reinforced clostridial medium (RCM) for routine growth and making competent cells, clostridial growth medium (CGM) for butanol challenge experiments [12]. Colonies were picked from agar-solidified plates at least 4 days old and were heat shocked at 80°C for 10 min before being used to inoculate cultures. In all experiments, growth in liquid medium was monitored by measuring the absorbance at 600 nm (*A*600) of appropriate dilutions with a UV/Vis 2802PC spectrophotometer (Unico, New Jersey, USA). For recombinant strains, antibiotics were added into the medium at the following final concentration: 100 µg/ml for ampicillin, 30 µg/ml for chloramphenicol and 50 µg/ml for erythromycin. All *C. acetobutylicum* and *E. coli* strains were stored at −80°C in RCM and L broth supplemented with 15% glycerol, respectively.

**Table 2:** Bacterial strains, plasmids and primers.

Strains, plasmids or primers	Relevant characteristics	Reference or source
**Strains**		
*E. coli* Top10	*mcrA Δ*(*mrr-hsdRMS-mcrBC*) *recA1*	Invitrogen
*E. coli* JM109	*recA1 mcrB^+^ hsdR17*	Lab storage
*C. acetobutylicum* DSM1731	Contains operon SMB_G1518-1519, wild type	DSMZ
*C. acetobutylicum* DDC14	ΔSMB_G1518-1519	22
*C. acetobutylicum* DC93	SMB_G1518::intron	This study
*C. acetobutylicum* DC94	SMB_G1519::intron	This study
1731(pIMP1)	DSM1731 containing plasmid pIMP1	This study
1731(p1518-1519)	DSM1731 containing plasmid p1518-1519	This study
**Plasmids**		
pMTL009	Cm^r^	35
pIMP1	MLS^r^ Amp^r^ shuttle vector of *E. coli*-*C. acetobutylicum*	24
pAN2	*Φ3t1*, *p15a ori*, Tet^r^, methylating DNA prior to transformation to protect it againsta *C. acetobutylicum* restriction system	36
pMTL009-1518	Derived from pMTL009, targeting the SMB_G1518 in *C. acetobutylicum*	This study
pMTL009-1519	Derived from pMTL009, targeting the SMB_G1519 in *C. acetobutylicum*	This study
		
p1518-1519	SMB_G1518-1519 expression vector	This study
**Primer**		
1518-160/161s-IBS	5′-AAAAAAGCTTATAATTATCCTTAAGGGGAAAGTATGTGCGCCCAGATAGGGTG	This study
1518-160/161s-EBS1d	5′-CAGATTGTACAAATGTGGTGATAACAGATAAGTCAAGTATGCTAACTTACCTTTCTTTGT	This study
1518-160/161s-EBS2	5′-TGAACGCAAGTTTCTAATTTCGGTTATACTTTCGATAGAGGAAAGTGTCT	This study
P1493-5	5′-ATGCCAAATGTGAAGTCTAT	This study
SMB_G1518-3E	5′-CTAAAATGTGCTTACACAAT	This study
Cac1494B	5?-TTGTGTAAGCACATTTTAGG	This study
Pex1494E	5?-TTATACACATATTGGCTCTC	This study
P1492	5′- ACGCGTCGACGACTTAAGGGAGACGAAGTC	This study
P1495-3E	5′-CCGGAATTCATCTCCTTCGCCTTCAGTTT	This study
Re-1493	5′-AGGAAGAGTGCTAAAGTTGTAG	This study
A2-14	5′-CTTGTTTGCCGATTTTACGAGA	This study

**Abbreviations**: Amp^r^, ampicillin resistance; Cm^r^, chloramphenicol resistance; Tet^r^, tetracycline resistance; *Φ3t1*, Φ3t1 methyltransferase gene of *Bacillus subtilis* phage Φ3t1. DSMZ, German Collection of Microorganisms and Cell Cultures, Braunschweig, Germany.

### The Disruption of SMB_G1518 and SMB_G1519

A group II intron based system modified by Dong was adopted to disrupt SMB_G1518 and SMB_G1519 [35]. Target sites in SMB_G1518 and SMB_G1519 for insertion were predicted in line with computer algorithm available at the Sigma-Aldrich website (www.sigmaaldrich.com/TargeTron Gene Knockout) and then the intron re-targeting PCR primers for SMB_G1518 including 1518-160/161s-IBS, 1518-160/161s-EBS1d and 1518-160/161s-EBS2 were designed, the primers for retargeting SMB_G1519 were recommended from the previous study (Table 2.) [22]. Disruption plasmids pMTL009-1518 and pMTL009-1519 were constructed and then introduced into DSM1731 followed the methods described by Dong and Heap, respectively [35], [36]. The verifying PCR primers for intron integrating into target sites of SMB_G1518 and SMB_G1519 were P1493-5 and SMB_G1518-3E, Cac1494B and Pex1494E (Table 2.). Genbank numbers of DC93 and DC94 was JN211186 and JN211187. For southern blot analysis of the disruption of SMB_G1518 and SMB_G1519, DNA probes CAC34 and Intron reported previously have been adopted in this study [22], [35].

### Overexpression of SMB_G1518-1519

A fragment from 387 bp upstream of SMB_G1518 (which includes the promoter of SMB_G1518) to 198 bp downstream of SMB_G1519 (a total of 1412 bp) was amplified by PCR from chromosomal DNA of *C. acetobutylicum* DSM 1731 with primers P1492 and P1495-3E (Table 2.). After double digestion, this fragment was ligated into SalI-EcoRI-linearized pIMP1 and verified by sequencing. The resulting plasmid was designated p1518-1519. Electrotransformation and screening for SMB_G1518-1519 overexpression strain followed the protocol developed by Mermelstein [37].

After cells were cultured with 1% (vol/vol) butanol for 6 h as described in butanol challenged experiment, RNA sampling and isolation were performed as previously described [12]. Complementary DNA (cDNA) was synthesized using a PrimeScriptTM 1^st^ Strand cDNA Synthesis Kit (TaKaRa Biotechnology (Dalian) Co., Ltd) with 1 µg of total RNA as the template. The primers Re-1493 and A2-14 used for the real-time PCR assay was designed targeting the junction between SMB_G1518 and SMB_G1519 (Table 2.). The 16S rRNA was used as the internal control for quantification and the primers were recommended from previous report [38]. PCR was carried out by Bio-Rad iQ5 Real-Time PCR Detection System (Bio-Rad Laboratories,Inc., Richmond, CA) in duplicates for at least three independent experiments with the following program: 3 min at 95°C, followed by 40 amplification cycles of 95°C for 20 s, 60°C for 20 s. The expression levels of SMB_G1518-1519 were normalized against the expression level of 16S rRNA. In addition, semi-quantitative PCR was adopted to compare the relative expression levels in the overexpression strain 1731(p1518-1519)and plasmid control strain 1731(pIMP1) using the cDNA as template.

### Butanol Challenge Experiments

Mutant and overexpression strains and their respective control strains were grown in 500 mL flasks containing 400 mL CGM at 37°C anaerobically. When the cell density attained *A*600 0.75±0.05, each culture was split into three 100 mL aliquots and then challenged with 0 or 1% (vol/vol) butanol, respectively. Effect of varied butanol concentrations on the growth of these strains was further measured by Unico UV-2000 Spectrophotometer. The concentration of glucose, acetate, butyrate, acetone, butanol and ethanol in broth cultures were determined followed the method described by Mao [38]. All experiments were performed in duplicate.

### Proteomics Sample Preparation

Cells were cultured with 1% (vol/vol) butanol for 6 h as described in butanol challenged experiment. Subsequent treatment of cells for proteomic analysis followed the methods described by Mao [38]. Protein concentration was measured by using 2-D Quant Kit (GE Healthcare, Uppsala, Sweden), and 1 mg aliquots were stored at −80°C.

### Comparative Proteomics Analysis

Two-dimensional gel electrophoresis (2-DE) was performed as described previously [38]. 2-DE analysis and protein identification were conducted with ImageMaster 6.0 2-D platinum analysis software and the Applied Biosystems 4700 Proteomics Analyzer MALDI-TOF/TOF (Applied Biosystems, Framingham, MA), followed the methods described by Mao [38]. For each condition, 2-DE experiments were carried out in triplicate.

### Motility Assays

*C. acetobutylicum* strains were grown in CGM at 37°C. After the cell density reached *A*600 0.75±0.05, 10 mL of culture was centrifuged and concentrated ten folds. 10 microliters of the concentrated cell suspension was spotted onto a CGM agar plate supplemented with 1% (vol/vol) butanol, CGM agar plate without butanol addition was used as the control. All plates were supplemented with 0.7% agar. The inoculated plates were incubated anaerobically for 48 h at 37°C. Photographs of the plates were taken with a Canon camera.

## Supporting Information

Figure S1 **Conservancy analysis of the region interacting with butanol in protein kinase C(PKC) α, δ.** A) Amino acid alignment of the C1A domains of PKC. B**)** Amino acid alignment of the C1B domains of PKC. Mus, *Mus musculus* (house mouse); Ory, *Oryctolagus cuniculus* (rabbit); Can, *Canis lupus* (dog); Rat, *Rattus norvegicus* (rat); Hom, *Homo sapiens* (human); Dro, *Drosophila melanogaster* (fruit fly); α, PKCα.
(TIF)

Figure S2 **Construction of SMB_G1518-1519 disruption mutants.** A) Two sets of primers P1493-5, SMB_G1518-3E and Cac1494B, Pex1494E flanking the target site of SMB_G1518 and SMB_G1519 were adopted to identify insertion mutants by PCR, The results showed that about 0.9-kb intron fragments were integrated into the target site of SMB_G1518 and SMB_G1519; B) SMB_G1518-1519 and the expected disrupted SMB_G1518 and SMB_G1519 in the chromosome were schematicly shown; C) Southern blot analysis of SMB_G1518 and SMB_G1519 disruption using CAC34 probe showed that the size of the CAC34-hybridized DNA fragments of strain DC93 and DC94 was about 0.9 kb larger than that of parental strain DSM 1731; D) Southern blot analysis of SMB_G1518 and SMB_G1519 disruption using Intron probe showed that no hybridized signals were detected in the lane of DSM 1731.
(TIF)

Figure S3 **Transcriptional analysis of SMB_G1518-1519.** A) Transcriptional analysis of SMB_G1518-1519 in DSM 1731, 1731(pIMP1) and 1731(p1518-1519) by Real-Time PCR; A, DSM 1731; B, 1731(pIMP1); C, 1731(p1518-1519). B) Transcriptional analysis of SMB_G1518-1519 in 1731(pIMP1) and 1731(p1518-1519) by semi-quantitative PCR; B1, 1731(pIMP1) under normal condition; B2, 1731(pIMP1) under butanol stress; C1, 1731(p1518-1519) under normal condition; C2, 1731(p1518-1519) under butanol stress; M, marker; N, negative control without DNA template.
(TIF)

Figure S4 **Images of all gels, DSM 1731 (left) and DDC14 (right) under normal condition.** a, b and c are experimental triplicate of each strain. Differentially expressed proteins are labeled, and details about them are shown in Table 1.
(TIF)

Figure S5 **Images of all gels, DSM 1731 (left) and DDC14 (right) under 1% butanol stress.** a, b and c are experimental triplicate of each strain. Differentially expressed proteins are labeled, and details about them are shown in Table 1.
(TIF)

Figure S6 **Images of all gels, 1731(pIMP1) (left) and 1731(p1518-1519) (right) under normal condition.** a, b and c are experimental triplicate of each strain. Differentially expressed proteins are labeled, and details about them are shown in Table 1.
(TIF)

Figure S7 **Images of all gels, 1731(pIMP1) (left) and 1731(p1518-1519) (right) under 1% butanol stress.** a, b and c are experimental triplicate of each strain. Differentially expressed proteins are labeled, and details about them are shown in Table 1.
(TIF)
