# Supplementary figures and images for: Identification and Characterization of Two Functionally Unknown Genes Involved in Butanol Tolerance of Clostridium acetobutylicum
Source: PLoS One. 2012 Jun 29;7(6):e38815. doi: 10.1371/journal.pone.0038815 (PMC3387226; doi:10.1371/journal.pone.0038815)

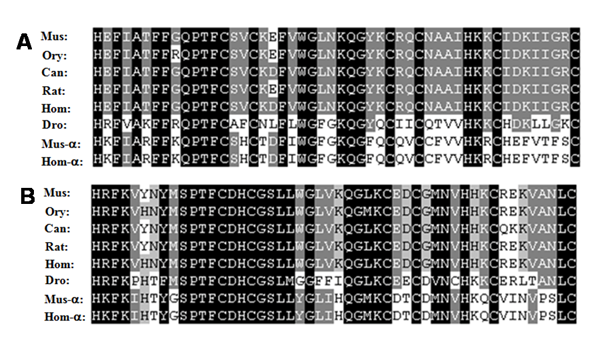

Supplement: Figure S1 — Conservancy analysis of the region interacting with butanol in protein kinase C(PKC) α, δ. A) Amino acid alignment of the C1A domains of PKC. B) Amino acid alignment of the C1B domains of PKC. Mus, Mus musculus (house mouse); Ory, Oryctolagus cuniculus (rabbit); Can, Canis lupus (dog); Rat, Rattus norvegicus (rat); Hom, Homo sapiens (human); Dro, Drosophila melanogaster (fruit fly); α, PKCα. (TIF) [file pone.0038815.s001.tif]

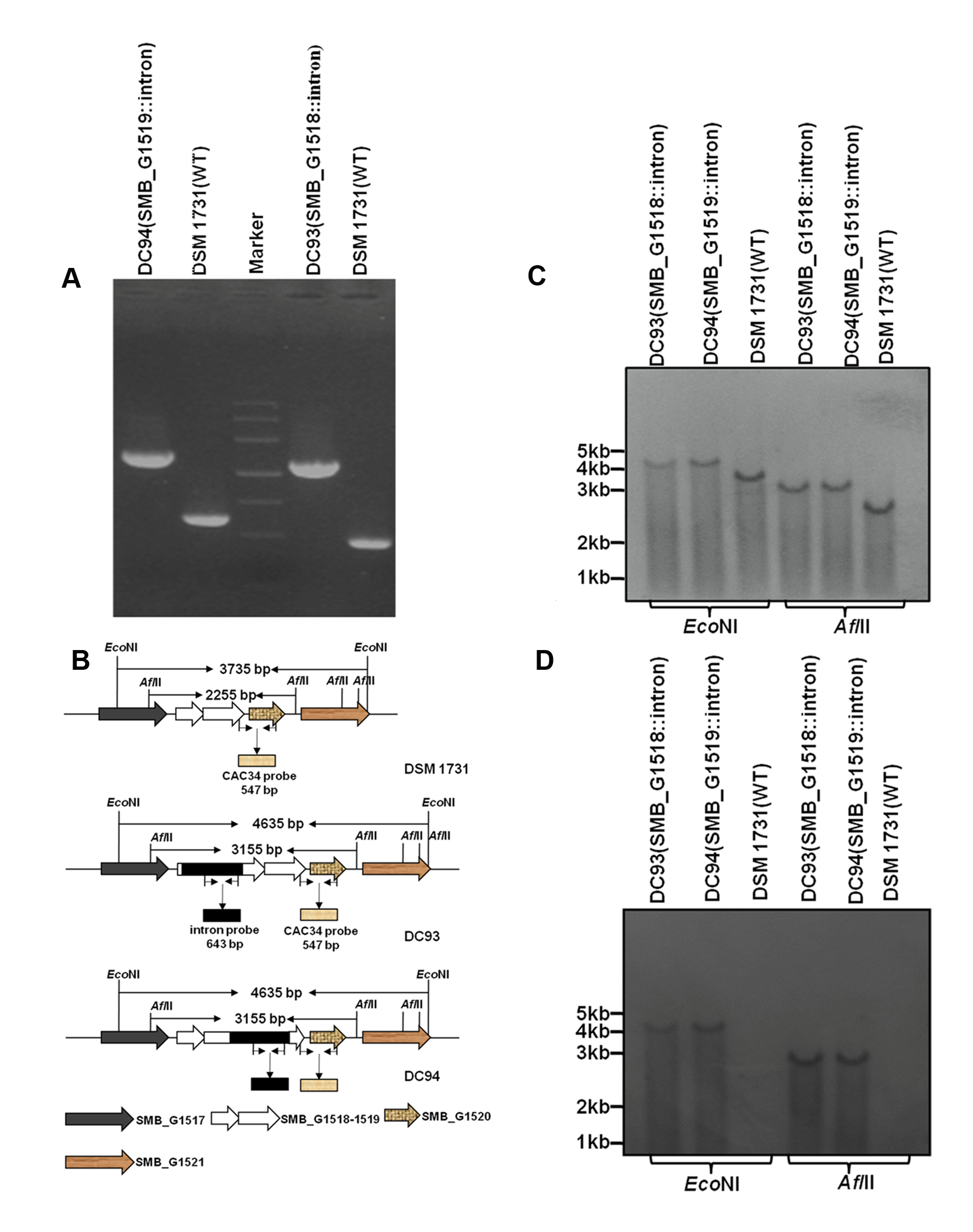

Supplement: Figure S2 — Construction of SMB_G1518-1519 disruption mutants. A) Two sets of primers P1493-5, SMB_G1518-3E and Cac1494B, Pex1494E flanking the target site of SMB_G1518 and SMB_G1519 were adopted to identify insertion mutants by PCR, The results showed that about 0.9-kb intron fragments were integrated into the target site of SMB_G1518 and SMB_G1519; B) SMB_G1518-1519 and the expected disrupted SMB_G1518 and SMB_G1519 in the chromosome were schematicly shown; C) Southern blot analysis of SMB_G1518 and SMB_G1519 disruption using CAC34 probe showed that the size of the CAC34-hybridized DNA fragments of strain DC93 and DC94 was about 0.9 kb larger than that of parental strain DSM 1731; D) Southern blot analysis of SMB_G1518 and SMB_G1519 disruption using Intron probe showed that no hybridized signals were detected in the lane of DSM 1731. (TIF) [file pone.0038815.s002.tif]

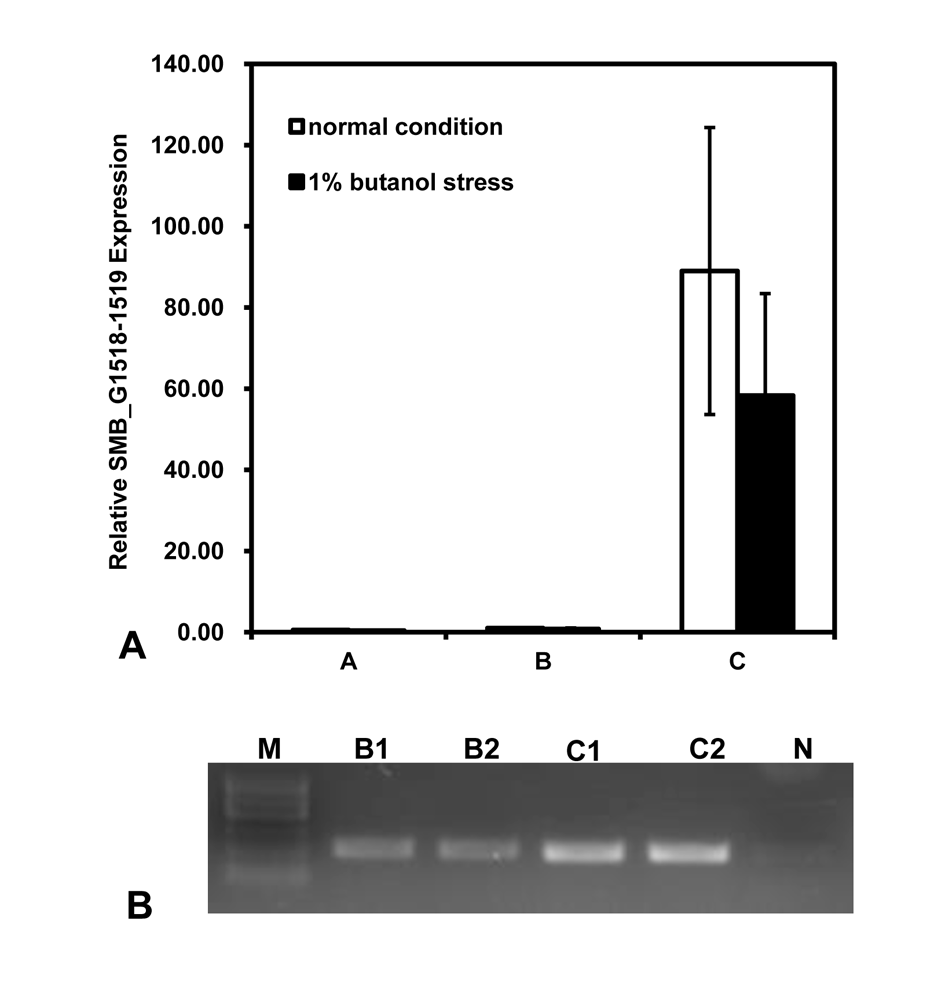

Supplement: Figure S3 — Transcriptional analysis of SMB_G1518-1519. A) Transcriptional analysis of SMB_G1518-1519 in DSM 1731, 1731(pIMP1) and 1731(p1518-1519) by Real-Time PCR; A, DSM 1731; B, 1731(pIMP1); C, 1731(p1518-1519). B) Transcriptional analysis of SMB_G1518-1519 in 1731(pIMP1) and 1731(p1518-1519) by semi-quantitative PCR; B1, 1731(pIMP1) under normal condition; B2, 1731(pIMP1) under butanol stress; C1, 1731(p1518-1519) under normal condition; C2, 1731(p1518-1519) under butanol stress; M, marker; N, negative control without DNA template. (TIF) [file pone.0038815.s003.tif]

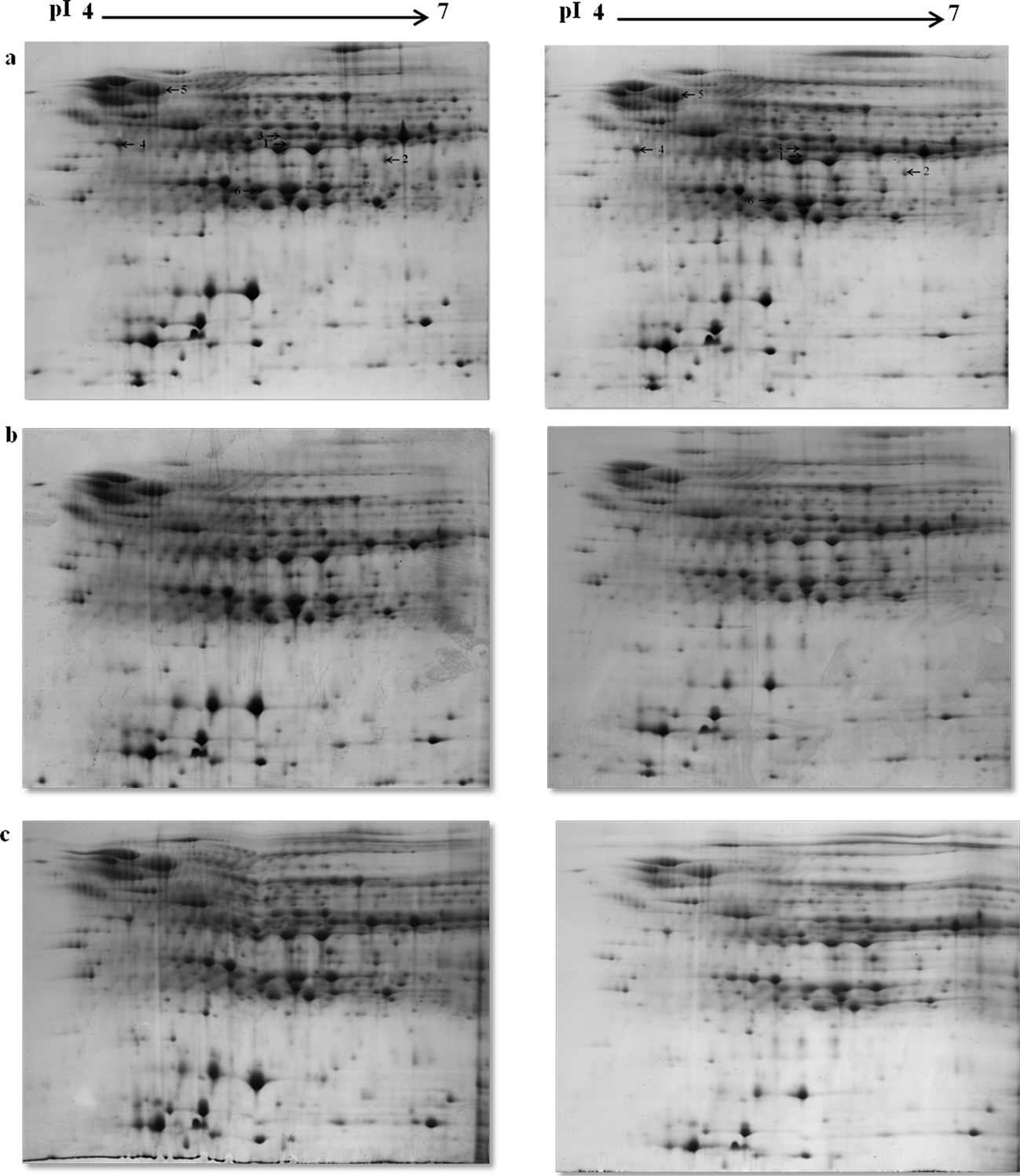

Supplement: Figure S4 — Images of all gels, DSM 1731 (left) and DDC14 (right) under normal condition. a, b and c are experimental triplicate of each strain. Differentially expressed proteins are labeled, and details about them are shown in Table 1. (TIF) [file pone.0038815.s004.tif]

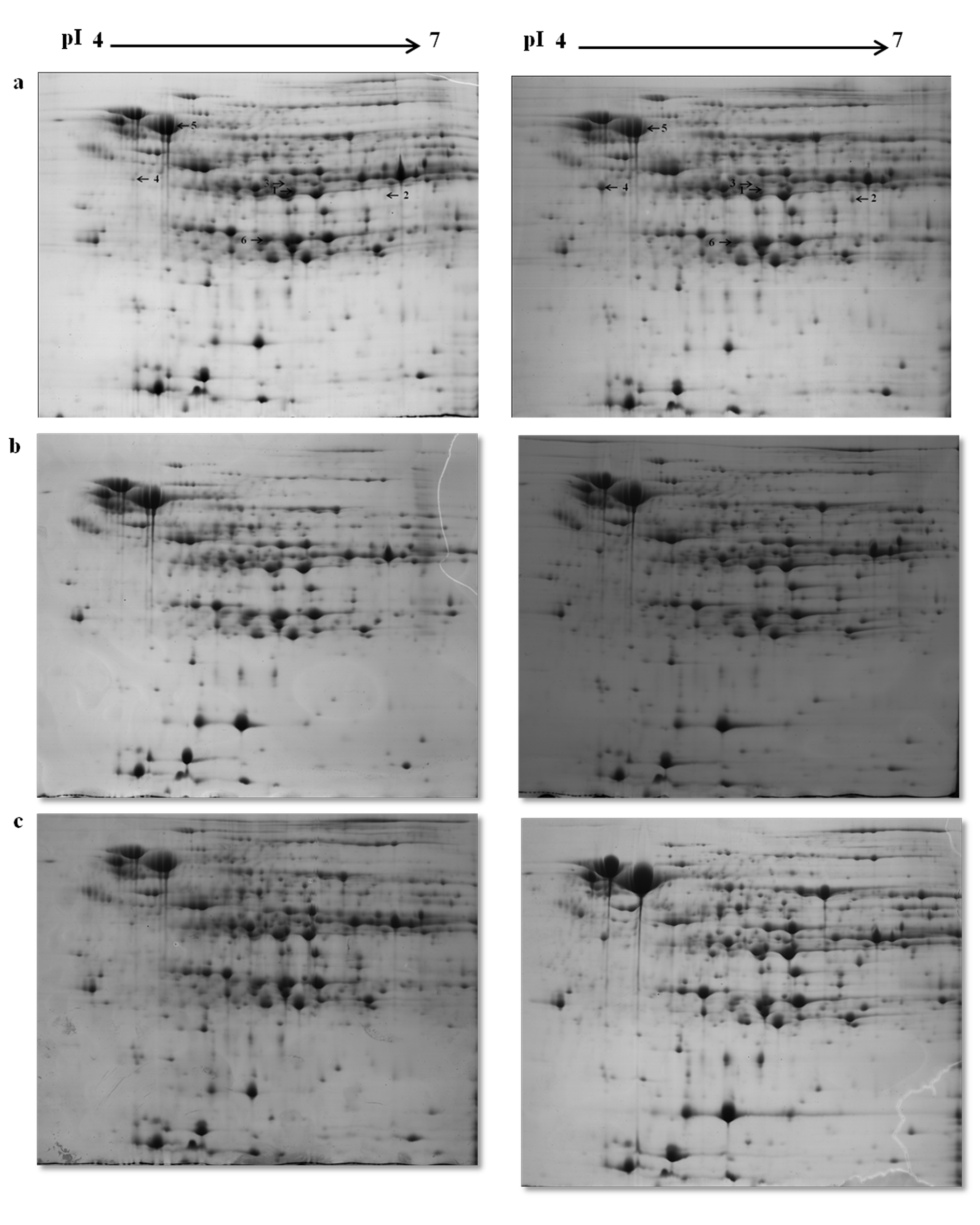

Supplement: Figure S5 — Images of all gels, DSM 1731 (left) and DDC14 (right) under 1% butanol stress. a, b and c are experimental triplicate of each strain. Differentially expressed proteins are labeled, and details about them are shown in Table 1. (TIF) [file pone.0038815.s005.tif]

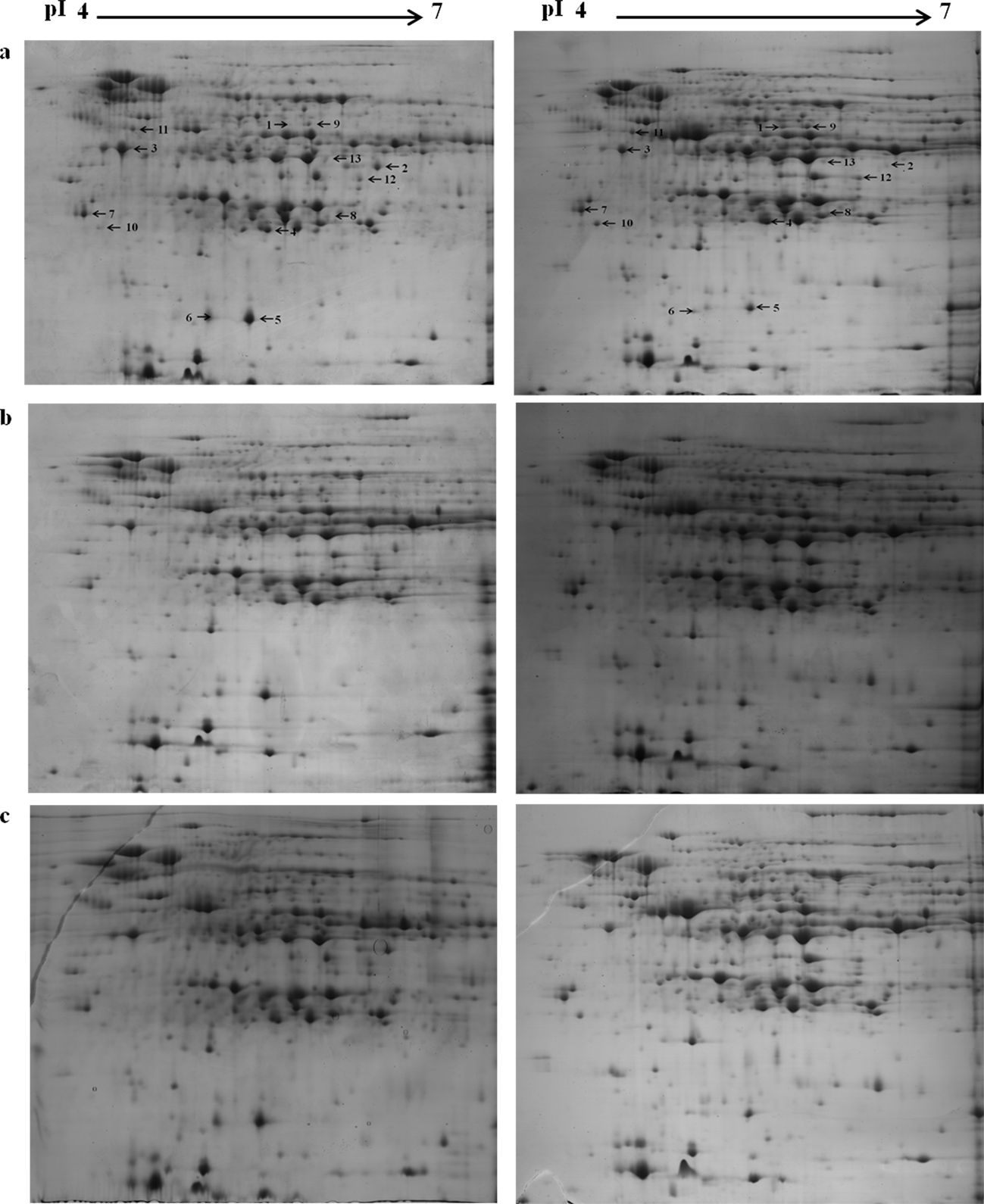

Supplement: Figure S6 — Images of all gels, 1731(pIMP1) (left) and 1731(p1518-1519) (right) under normal condition. a, b and c are experimental triplicate of each strain. Differentially expressed proteins are labeled, and details about them are shown in Table 1. (TIF) [file pone.0038815.s006.tif]

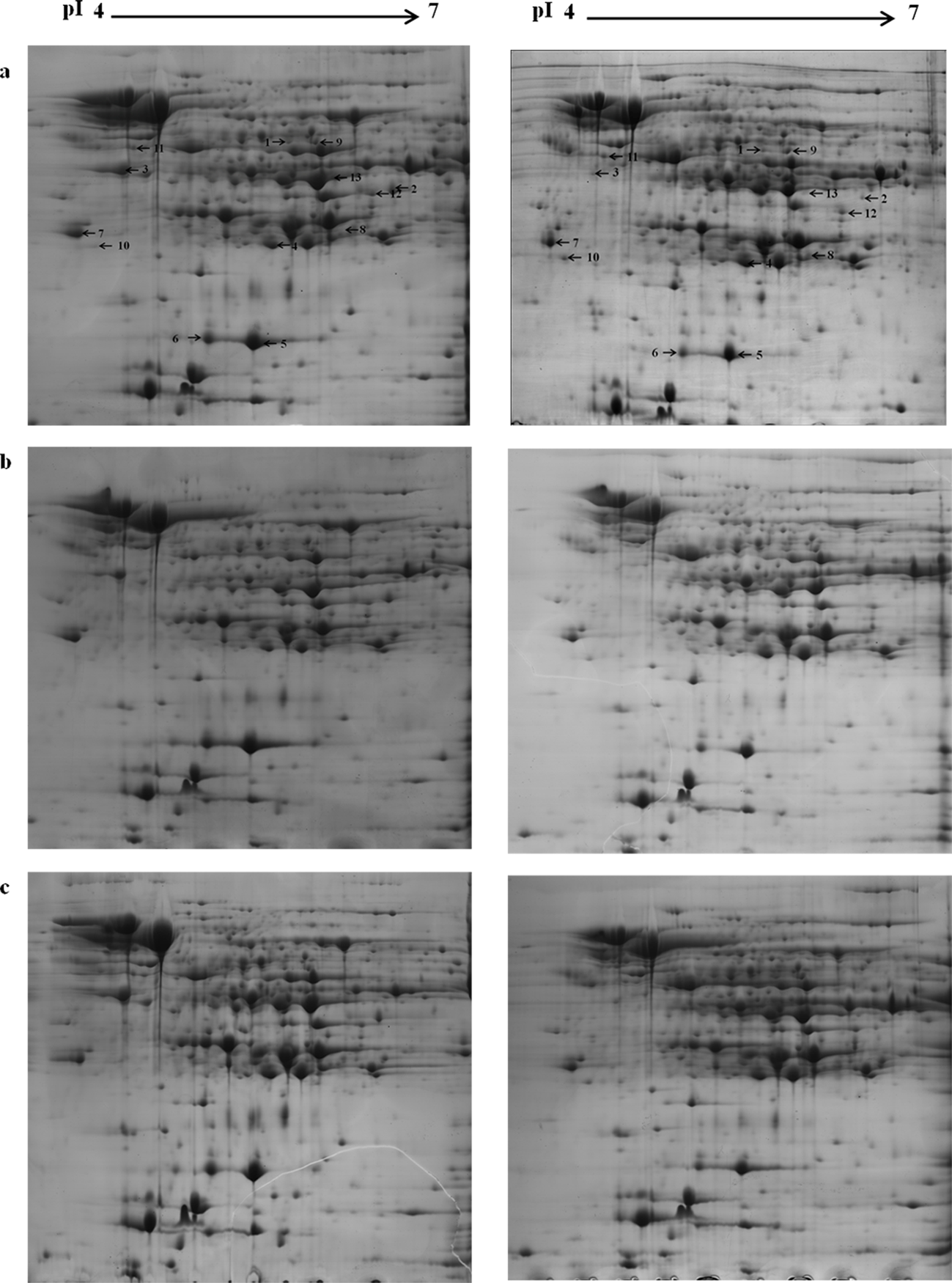

Supplement: Figure S7 — Images of all gels, 1731(pIMP1) (left) and 1731(p1518-1519) (right) under 1% butanol stress. a, b and c are experimental triplicate of each strain. Differentially expressed proteins are labeled, and details about them are shown in Table 1. (TIF) [file pone.0038815.s007.tif]
